# 4-Chloro­curcumin

**DOI:** 10.1107/S2414314624012434

**Published:** 2025-01-03

**Authors:** Phuong-Truc T. Pham, Mamoun M. Bader

**Affiliations:** a Department of Chemistry Pennsylvania State Scranton Dunmore PA 18512 USA; b Department of Chemistry Alfaisal University,Riyadh 11533 Saudi Arabia; University of Aberdeen, United Kingdom

**Keywords:** crystal structure, curcumin derivative, hydrogen bonds

## Abstract

The title compound [systematic name: 4-chloro-5-hy­droxy-1,7-bis­(4-hy­droxy-3-meth­oxy­phen­yl)hepta-1,4,6-trien-3-one], C_21_H_19_ClO_6_, is close to planar, with a dihedral angle of 2.61 (7)° between the terminal phenyl groups and three intra­molecular O—H⋯O hydrogen bonds occur. In the crystal, the mol­ecules are linked into [201] chains by O—H⋯O hydrogen bonds and weak aromatic π–π stacking is also observed with a shortest centroid–centroid separation of 3.7279 (8) Å.

## Abstract 2

Intra- and inter-mol­ecular O—H⋯O hydrogen bonds are observed in the structure of the title compound.

## Graphical abstract

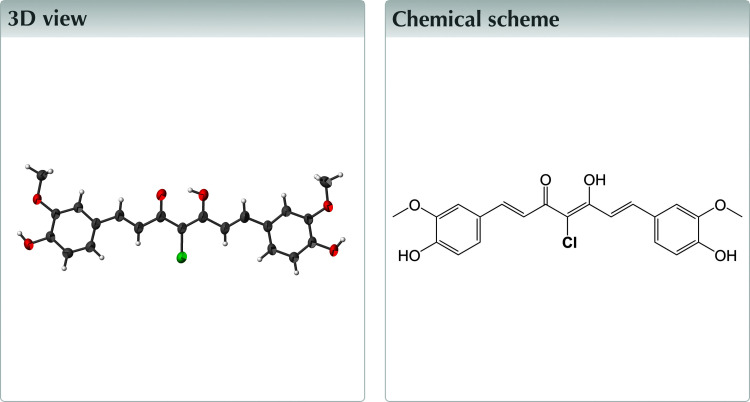

## Structure description

Curcumin, or 1,7-bis­(4-hy­droxy-3-meth­oxy­phen­yl)-1,6-hepta­diene-3,5-dione (C_21_H_20_O_6_), is a yellow–orange polyphenolic compound found in turmeric. Since the 1990s, extensive research has highlighted its anti­oxidant, anti-inflammatory, and anti­cancer properties (Dairam *et al.*, 2008 ▸). Structurally, curcumin features an α,β-unsaturated β-diketone moiety. In neutral and acidic media, it predominantly adopts the diketo form, whereas the more stable keto–enol form is favored under alkaline conditions. The phenolic groups and the α,β-unsaturated diketone contribute to its anti­oxidant activity, while the α,β-unsaturated diketone unit is primarily linked to its anti­cancer effects (Priyadarsini, 2013 ▸). An examination of the Cambridge Structural Database (CSD; version2024.3, update of December 2024; Groom *et al.*, 2016 ▸) indicates that curcumin exists in three polymorphs (I, II, and III), all displaying their keto–enol tautomeric forms in the solid-state. The most common form, polymorph I, crystallizes in the monoclinic space group *P2*/*n* [CSD refcodes BINMEQ (Tønnesen *et al.*, 1982 ▸), BINMEQ01 (Ishigami *et al.*, 1999 ▸), BINMEQ02 (Parimita *et al.*, 2007 ▸), BINMEQ03 (Suo *et al.*, 2006 ▸), BINMEQ04 (Fronczek, 2009 ▸), BINMEQ05 (Sanphui *et al.*, 2011 ▸), BINMEQ09 (Reid *et al.*, 2015 ▸), BINMEQ10 (Parveen *et al.*, 2016 ▸), BINMEQ11 (Matlinska *et al.*, 2018 ▸), BINMEQ13 (Lal *et al.*, 2020 ▸) and BINMEQ14 (Kohnhorst & Saithong, 2019 ▸)] while the less common forms II and III crystallize in the ortho­rhom­bic space groups *Pca*2_1_ [BINMEQ06 (Sanphui *et al.*, 2011 ▸), BINMEQ08 (Renuga Parameswari *et al.*, 2012 ▸), BINMEQ12 (Matlinska *et al.*, 2018 ▸) and BINMEQ15 (Zou, 2024 ▸)] and *Pbca* (BINMEQ07; Sanphui *et al.*, 2011 ▸), respectively.

This study presents the synthesis and crystal structure of the title compound, C_21_H_19_ClO_6_ (**I**), where the hydrogen atom at the α-carbon atom (4-position) is replaced by a chlorine atom. The synthesis of the title compound was reported previously by two groups through multistep syntheses plagued with low yields and impurities (Ooko *et al.*, 2016 ▸; Abood *et al.*, 2021 ▸). Our method is a direct one-step halogenation reaction with a reasonable yield.

**Figure 1:**
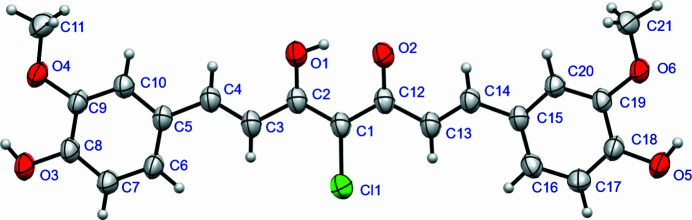
The mol­ecular structure of (**I**) showing 50% displacement ellipsoids. Hydrogen bonds are shown as dotted lines.

The mol­ecule of (**I**) adopts a near planar conformation, as indicated by the torsion angle of 2.61 (7)(7)° between the planes of the terminal C5–C10 and C15–C20 phenyl groups. Three intra­molecular O—H⋯O hydrogen bonds occur (Fig. 1 ▸), with the central O1—H1⋯O2 bond notably shorter and closer to linearity than the terminal O3—H3⋯O4 and O5—H5⋯O6 bonds (Table 1 ▸). The supporting information provides a comparison of curcumin polymorph structural and physical data with those of (**I**).

**Figure 2:**
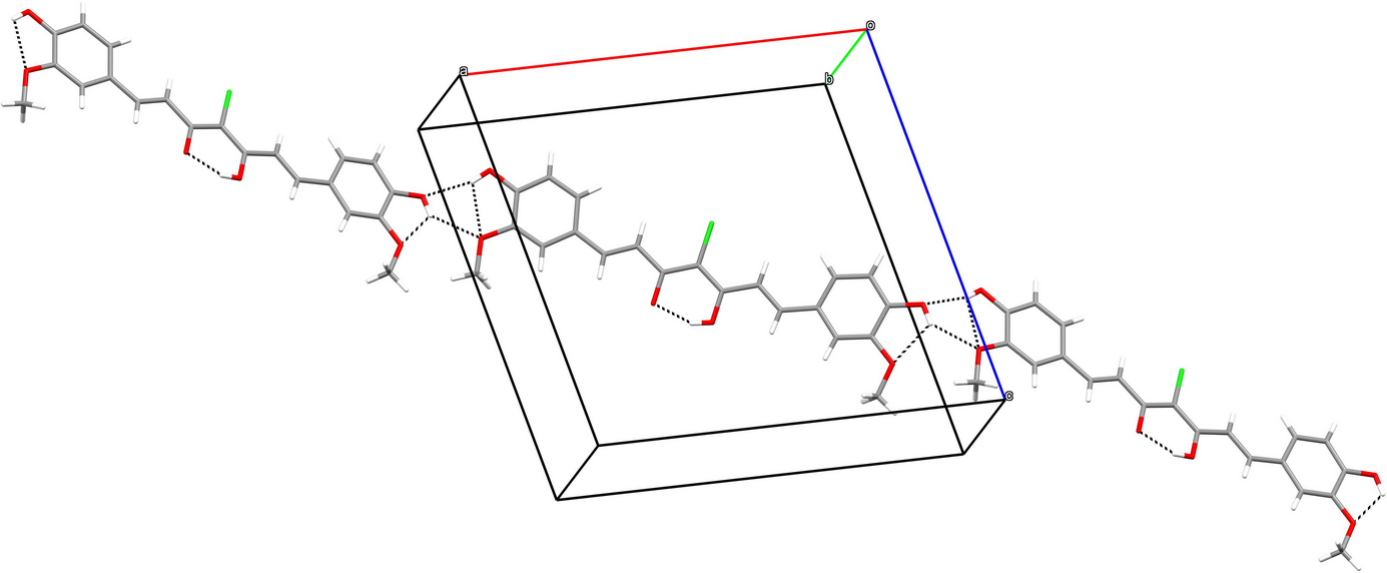
Part of a [20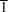] hydrogen-bonded chain in the structure of (**I**).

In the crystal of (**I**), the mol­ecules are linked by O—H⋯O hydrogen bonds arising from O3 and O4 (both of which also form an intra­molecular link) to generate infinite [20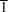] chains (Fig. 2 ▸). Aromatic π–π stacking also occurs, as indicated by the shortest centroid–centroid separation of 3.7279 (8) Å between inversion related C5–C10 and C15–C20 rings but no short Cl⋯Cl contacts occur.

## Synthesis and crystallization

Curcumin (2.74 g, 7.45 mmol) was dissolved in anhydrous aceto­nitrile with heating. The solution was briefly cooled in an ice bath before *N*-chloro­succinimide (1.21 g, 9.05 mmol) was added. Stirring was allowed to continue overnight at room temperature. The red crude powder was filtered and recrystallized from aceto­nitrile solution to give yellow needles of (**I**) (yield: 33%. Analysis calculated (C_21_H_19_ClO_6_): C, 62.62; H, 4.75; Cl, 8.80. Found: C, 62.30; H, 4.77; Cl, 8.58. Exact mass: 402.0870, found (EI, *M* + 1): 403.0940. M.p. 197°C (lit. 190–191°C; Abood *et al.*, 2021 ▸). Compared to curcumin, the solubility of (**I**) in water is slightly reduced, measuring approximately 5 g l^−1^, compared to 6.6 g l^−1^ for the former.

**Figure 3:**
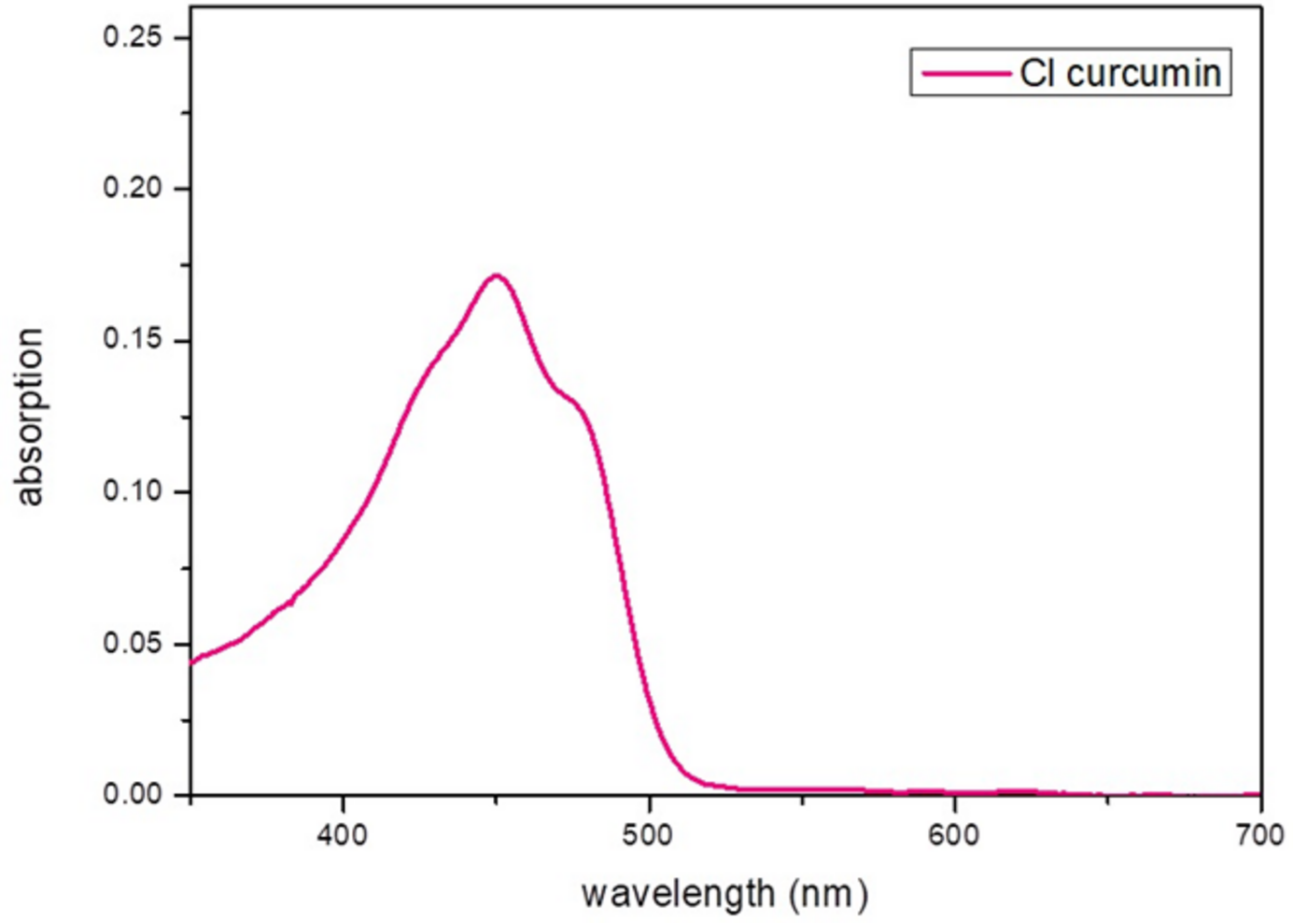
The UV/visible absorption spectrum of (**I**) dissolved in di­chloro­methane.

The UV/visible absorption spectrum of (**I**) dissolved in di­chloro­methane shows a bathochromic (red) shift of 35 nm, compared to the parent curcumin compound (Fig. 3 ▸), which might correlate with the electron-donating properties of the chlorine atom.

## Refinement

Crystal data, data collection and structure refinement details are summarized in Table 2 ▸.

## Supplementary Material

Crystal structure: contains datablock(s) I. DOI: 10.1107/S2414314624012434/hb4501sup1.cif

Structure factors: contains datablock(s) I. DOI: 10.1107/S2414314624012434/hb4501Isup2.hkl

supporting information. DOI: 10.1107/S2414314624012434/hb4501sup3.doc

Supporting information file. DOI: 10.1107/S2414314624012434/hb4501Isup4.cml

CCDC reference: 2258982

Additional supporting information:  crystallographic information; 3D view; checkCIF report

## Figures and Tables

**Table 1:** Hydrogen-bond geometry (Å, °)

*D*—H⋯*A*	*D*—H	H⋯*A*	*D*⋯*A*	*D*—H⋯*A*
O1—H1⋯O2	0.82	1.70	2.4506 (16)	151
O3—H3⋯O4	0.82	2.18	2.6395 (15)	115
O5—H5⋯O6	0.82	2.28	2.7200 (15)	114
O3—H3⋯O6^i^	0.82	2.20	2.8398 (15)	135
O5—H5⋯O3^ii^	0.82	2.05	2.8439 (16)	164
C11—H11*B*⋯O2^iii^	0.96	2.59	3.477 (2)	154
C17—H17⋯O2^iv^	0.93	2.57	3.3206 (19)	138

Symmetry codes: (i) 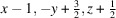; (ii) 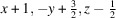; (iii) 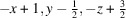; (iv) 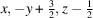.

**Table 2:** Experimental details

Crystal data	
Chemical formula	C_21_H_19_ClO_6_
*M*_r_	402.81
Crystal system, space group	Monoclinic, *P*2_1_/*c*
Temperature (K)	298
*a*, *b*, *c* (Å)	16.7520 (3), 7.27831 (16), 15.9369 (3)
β (°)	100.0131 (17)
*V* (Å^3^)	1913.53 (7)
*Z*	4
Radiation type	Cu *K*α
μ (mm^−1^)	2.08
Crystal size (mm)	0.2 × 0.17 × 0.13

Data collection	
Diffractometer	Four-circle diffractometer
Absorption correction	Multi-scan (*CrysAlis PRO*; Rigaku OD, 2022 ▸)
*T*_min_, *T*_max_	0.765, 1.000
No. of measured, independent and observed [*I* > 2σ(*I*)] reflections	13358, 3710, 3247
*R*_int_	0.020
(sin θ/λ)_max_ (Å^−1^)	0.623

Refinement	
*R*[*F*^2^ > 2σ(*F*^2^)], *wR*(*F*^2^), *S*	0.036, 0.108, 1.07
No. of reflections	3710
No. of parameters	259
H-atom treatment	H-atom parameters constrained
Δρ_max_, Δρ_min_ (e Å^−3^)	0.27, −0.32

Computer programs: *CrysAlis PRO* (Rigaku OD, 2022 ▸), *SHELXT* (Sheldrick, 2015*a* ▸), *SHELXL2018/3* (Sheldrick, 2015*b* ▸) and *OLEX2* (Dolomanov *et al.*, 2009 ▸).

## full crystallographic data

### 4-Chloro-5-hydroxy-1,7-bis(4-hydroxy-3-methoxyphenyl)hepta-1,4,6-trien-3-one Crystal data

**Table:**

C_21_H_19_ClO_6_	*F*(000) = 840
*M**_r_* = 402.81	*D*_x_ = 1.398 Mg m^−^^3^
Monoclinic, *P*2_1_/*c*	Cu *K*α radiation, λ = 1.54184 Å
*a* = 16.7520 (3) Å	Cell parameters from 8805 reflections
*b* = 7.27831 (16) Å	θ = 2.9–73.7°
*c* = 15.9369 (3) Å	µ = 2.08 mm^−^^1^
β = 100.0131 (17)°	*T* = 298 K
*V* = 1913.53 (7) Å^3^	Block, clear orange
*Z* = 4	0.2 × 0.17 × 0.13 mm

### 4-Chloro-5-hydroxy-1,7-bis(4-hydroxy-3-methoxyphenyl)hepta-1,4,6-trien-3-one Data collection

**Table:**

Four-circle diffractometer	3710 independent reflections
Radiation source: Rotating-anode X-ray tube, Rigaku (Cu) X-ray Source	3247 reflections with *I* > 2σ(*I*)
Mirror monochromator	*R*_int_ = 0.020
Detector resolution: 10.0000 pixels mm^-1^	θ_max_ = 73.8°, θ_min_ = 2.7°
ω scans	*h* = −20→18
Absorption correction: multi-scan (CrysAlisPro; Rigaku OD, 2022)	*k* = −8→8
*T*_min_ = 0.765, *T*_max_ = 1.000	*l* = −18→19
13358 measured reflections	

### 4-Chloro-5-hydroxy-1,7-bis(4-hydroxy-3-methoxyphenyl)hepta-1,4,6-trien-3-one Refinement

**Table:**

Refinement on *F*^2^	Hydrogen site location: inferred from neighbouring sites
Least-squares matrix: full	H-atom parameters constrained
*R*[*F*^2^ > 2σ(*F*^2^)] = 0.036	*w* = 1/[σ^2^(*F*_o_^2^) + (0.0595*P*)^2^ + 0.3344*P*] where *P* = (*F*_o_^2^ + 2*F*_c_^2^)/3
*wR*(*F*^2^) = 0.108	(Δ/σ)_max_ < 0.001
*S* = 1.07	Δρ_max_ = 0.27 e Å^−^^3^
3710 reflections	Δρ_min_ = −0.32 e Å^−^^3^
259 parameters	Extinction correction: *SHELXL2018/3* (Sheldrick, 2015b), Fc^*^=kFc[1+0.001xFc^2^λ^3^/sin(2θ)]^-1/4^
0 restraints	Extinction coefficient: 0.0016 (3)
Primary atom site location: dual	

### 4-Chloro-5-hydroxy-1,7-bis(4-hydroxy-3-methoxyphenyl)hepta-1,4,6-trien-3-one Special details

**Table:**

Geometry. All esds (except the esd in the dihedral angle between two l.s. planes) are estimated using the full covariance matrix. The cell esds are taken into account individually in the estimation of esds in distances, angles and torsion angles; correlations between esds in cell parameters are only used when they are defined by crystal symmetry. An approximate (isotropic) treatment of cell esds is used for estimating esds involving l.s. planes.

### 4-Chloro-5-hydroxy-1,7-bis(4-hydroxy-3-methoxyphenyl)hepta-1,4,6-trien-3-one Fractional atomic coordinates and isotropic or equivalent isotropic displacement parameters (Å^2^)

**Table:**

	*x*	*y*	*z*	*U*_iso_*/*U*_eq_	
Cl1	0.43756 (2)	0.67682 (7)	0.37034 (2)	0.06172 (16)	
O1	0.50652 (7)	0.79498 (18)	0.61198 (7)	0.0570 (3)	
H1	0.550936	0.829843	0.603374	0.086*	
O2	0.61937 (6)	0.85947 (18)	0.53705 (7)	0.0565 (3)	
O3	0.04255 (6)	0.4600 (2)	0.66977 (7)	0.0641 (4)	
H3	0.041337	0.463367	0.720969	0.096*	
O4	0.15138 (7)	0.56430 (19)	0.80133 (6)	0.0581 (3)	
O5	0.87723 (7)	0.9296 (2)	0.13546 (7)	0.0682 (4)	
H5	0.922848	0.967388	0.154746	0.102*	
O6	0.94585 (6)	1.00942 (19)	0.29885 (6)	0.0573 (3)	
C1	0.49645 (8)	0.7455 (2)	0.46575 (9)	0.0434 (3)	
C2	0.46339 (8)	0.7392 (2)	0.54077 (9)	0.0443 (3)	
C3	0.38188 (9)	0.6737 (2)	0.54378 (10)	0.0477 (4)	
H3A	0.349488	0.633170	0.493829	0.057*	
C4	0.35239 (9)	0.6703 (2)	0.61630 (10)	0.0474 (4)	
H4	0.387375	0.707950	0.665095	0.057*	
C5	0.27120 (8)	0.6139 (2)	0.62761 (9)	0.0426 (3)	
C6	0.21122 (9)	0.5600 (2)	0.56049 (9)	0.0476 (4)	
H6	0.222603	0.557438	0.505431	0.057*	
C7	0.13493 (9)	0.5102 (2)	0.57431 (9)	0.0508 (4)	
H7	0.095275	0.475143	0.528788	0.061*	
C8	0.11786 (8)	0.5126 (2)	0.65602 (9)	0.0459 (3)	
C9	0.17684 (8)	0.5667 (2)	0.72415 (9)	0.0437 (3)	
C10	0.25293 (9)	0.6168 (2)	0.71009 (9)	0.0451 (3)	
H10	0.292303	0.652633	0.755702	0.054*	
C11	0.20933 (11)	0.6084 (3)	0.87479 (10)	0.0649 (5)	
H11A	0.229870	0.730055	0.868938	0.097*	
H11B	0.253195	0.521893	0.880651	0.097*	
H11C	0.184072	0.603208	0.924386	0.097*	
C12	0.57657 (9)	0.8078 (2)	0.46588 (9)	0.0435 (3)	
C13	0.61302 (9)	0.8162 (2)	0.38921 (10)	0.0464 (4)	
H13	0.582583	0.783486	0.336848	0.056*	
C14	0.68960 (9)	0.8704 (2)	0.39371 (10)	0.0453 (3)	
H14	0.716934	0.901139	0.447769	0.054*	
C15	0.73604 (8)	0.8876 (2)	0.32490 (9)	0.0423 (3)	
C16	0.70374 (9)	0.8547 (2)	0.24003 (10)	0.0487 (4)	
H16	0.649540	0.821538	0.224812	0.058*	
C17	0.75172 (9)	0.8709 (2)	0.17775 (10)	0.0521 (4)	
H17	0.729421	0.848662	0.121046	0.063*	
C18	0.83262 (9)	0.9199 (2)	0.19931 (9)	0.0471 (3)	
C19	0.86553 (8)	0.9572 (2)	0.28391 (9)	0.0434 (3)	
C20	0.81761 (8)	0.9398 (2)	0.34580 (9)	0.0442 (3)	
H20	0.839899	0.963208	0.402395	0.053*	
C21	0.98395 (10)	1.0319 (3)	0.38498 (10)	0.0639 (5)	
H21A	1.038542	1.074016	0.386889	0.096*	
H21B	0.984627	0.916388	0.414174	0.096*	
H21C	0.954454	1.120490	0.412132	0.096*	

### 4-Chloro-5-hydroxy-1,7-bis(4-hydroxy-3-methoxyphenyl)hepta-1,4,6-trien-3-one Atomic displacement parameters (Å^2^)

**Table:**

	*U*^11^	*U*^22^	*U*^33^	*U*^12^	*U*^13^	*U*^23^
Cl1	0.0402 (2)	0.0935 (4)	0.0527 (2)	−0.01305 (19)	0.01165 (17)	−0.00591 (19)
O1	0.0407 (6)	0.0814 (8)	0.0512 (6)	−0.0087 (6)	0.0141 (5)	0.0033 (5)
O2	0.0340 (5)	0.0848 (8)	0.0516 (6)	−0.0076 (5)	0.0097 (4)	0.0013 (6)
O3	0.0334 (6)	0.1104 (10)	0.0526 (6)	−0.0065 (6)	0.0187 (5)	−0.0016 (7)
O4	0.0452 (6)	0.0933 (9)	0.0395 (5)	−0.0025 (6)	0.0175 (4)	−0.0007 (5)
O5	0.0469 (6)	0.1209 (11)	0.0400 (6)	−0.0127 (7)	0.0165 (5)	0.0010 (6)
O6	0.0313 (5)	0.1007 (10)	0.0419 (5)	−0.0091 (5)	0.0118 (4)	0.0003 (5)
C1	0.0314 (7)	0.0511 (8)	0.0493 (7)	0.0004 (6)	0.0113 (6)	0.0058 (6)
C2	0.0343 (7)	0.0491 (8)	0.0514 (8)	0.0015 (6)	0.0124 (6)	0.0067 (6)
C3	0.0354 (7)	0.0552 (9)	0.0553 (8)	−0.0011 (6)	0.0163 (6)	0.0049 (7)
C4	0.0391 (8)	0.0542 (9)	0.0514 (8)	−0.0025 (6)	0.0151 (6)	0.0035 (6)
C5	0.0371 (7)	0.0481 (8)	0.0456 (7)	0.0016 (6)	0.0151 (6)	0.0043 (6)
C6	0.0417 (8)	0.0636 (9)	0.0415 (7)	0.0011 (7)	0.0179 (6)	0.0032 (6)
C7	0.0367 (7)	0.0748 (11)	0.0420 (7)	−0.0007 (7)	0.0100 (6)	−0.0012 (7)
C8	0.0310 (7)	0.0631 (9)	0.0464 (7)	0.0014 (6)	0.0144 (6)	0.0019 (7)
C9	0.0378 (7)	0.0563 (9)	0.0399 (7)	0.0045 (6)	0.0145 (6)	0.0032 (6)
C10	0.0377 (7)	0.0554 (8)	0.0439 (7)	−0.0017 (6)	0.0116 (6)	0.0001 (6)
C11	0.0625 (11)	0.0925 (14)	0.0404 (8)	0.0013 (10)	0.0106 (7)	−0.0003 (8)
C12	0.0323 (7)	0.0490 (8)	0.0505 (8)	0.0029 (6)	0.0109 (6)	0.0071 (6)
C13	0.0354 (7)	0.0542 (9)	0.0521 (8)	0.0003 (6)	0.0147 (6)	0.0036 (6)
C14	0.0354 (7)	0.0538 (9)	0.0493 (8)	0.0007 (6)	0.0145 (6)	0.0014 (6)
C15	0.0334 (7)	0.0470 (8)	0.0488 (8)	0.0012 (6)	0.0131 (6)	0.0034 (6)
C16	0.0337 (7)	0.0605 (9)	0.0519 (8)	−0.0048 (6)	0.0076 (6)	0.0048 (7)
C17	0.0434 (8)	0.0711 (10)	0.0407 (7)	−0.0062 (7)	0.0041 (6)	0.0037 (7)
C18	0.0405 (7)	0.0633 (9)	0.0397 (7)	0.0007 (7)	0.0136 (6)	0.0057 (6)
C19	0.0300 (7)	0.0587 (9)	0.0427 (7)	0.0003 (6)	0.0099 (5)	0.0043 (6)
C20	0.0340 (7)	0.0596 (9)	0.0403 (7)	−0.0007 (6)	0.0098 (5)	0.0003 (6)
C21	0.0372 (8)	0.1081 (15)	0.0465 (8)	−0.0085 (9)	0.0072 (6)	−0.0080 (9)

### 4-Chloro-5-hydroxy-1,7-bis(4-hydroxy-3-methoxyphenyl)hepta-1,4,6-trien-3-one Geometric parameters (Å, º)

**Table:**

Cl1—C1	1.7359 (15)	C7—C8	1.381 (2)
O1—H1	0.8200	C8—C9	1.392 (2)
O1—C2	1.2999 (18)	C9—C10	1.381 (2)
O2—C12	1.2881 (19)	C10—H10	0.9300
O3—H3	0.8200	C11—H11A	0.9600
O3—C8	1.3718 (17)	C11—H11B	0.9600
O4—C9	1.3703 (16)	C11—H11C	0.9600
O4—C11	1.422 (2)	C12—C13	1.459 (2)
O5—H5	0.8200	C13—H13	0.9300
O5—C18	1.3650 (17)	C13—C14	1.332 (2)
O6—C19	1.3785 (16)	C14—H14	0.9300
O6—C21	1.4190 (18)	C14—C15	1.4561 (19)
C1—C2	1.4030 (19)	C15—C16	1.387 (2)
C1—C12	1.4164 (19)	C15—C20	1.4016 (19)
C2—C3	1.4550 (19)	C16—H16	0.9300
C3—H3A	0.9300	C16—C17	1.387 (2)
C3—C4	1.334 (2)	C17—H17	0.9300
C4—H4	0.9300	C17—C18	1.386 (2)
C4—C5	1.4614 (19)	C18—C19	1.392 (2)
C5—C6	1.391 (2)	C19—C20	1.3814 (19)
C5—C10	1.4007 (19)	C20—H20	0.9300
C6—H6	0.9300	C21—H21A	0.9600
C6—C7	1.383 (2)	C21—H21B	0.9600
C7—H7	0.9300	C21—H21C	0.9600
			
C2—O1—H1	109.5	O4—C11—H11C	109.5
C8—O3—H3	109.5	H11A—C11—H11B	109.5
C9—O4—C11	117.50 (12)	H11A—C11—H11C	109.5
C18—O5—H5	109.5	H11B—C11—H11C	109.5
C19—O6—C21	117.40 (11)	O2—C12—C1	118.50 (13)
C2—C1—Cl1	119.25 (11)	O2—C12—C13	118.38 (13)
C2—C1—C12	121.57 (13)	C1—C12—C13	123.13 (14)
C12—C1—Cl1	119.18 (11)	C12—C13—H13	119.8
O1—C2—C1	119.47 (13)	C14—C13—C12	120.44 (14)
O1—C2—C3	117.12 (13)	C14—C13—H13	119.8
C1—C2—C3	123.41 (14)	C13—C14—H14	115.8
C2—C3—H3A	119.1	C13—C14—C15	128.36 (15)
C4—C3—C2	121.73 (15)	C15—C14—H14	115.8
C4—C3—H3A	119.1	C16—C15—C14	123.40 (13)
C3—C4—H4	116.4	C16—C15—C20	118.51 (12)
C3—C4—C5	127.22 (15)	C20—C15—C14	118.09 (13)
C5—C4—H4	116.4	C15—C16—H16	119.8
C6—C5—C4	123.22 (13)	C17—C16—C15	120.45 (14)
C6—C5—C10	118.65 (13)	C17—C16—H16	119.8
C10—C5—C4	118.13 (13)	C16—C17—H17	119.7
C5—C6—H6	119.5	C18—C17—C16	120.54 (14)
C7—C6—C5	121.05 (13)	C18—C17—H17	119.7
C7—C6—H6	119.5	O5—C18—C17	117.85 (13)
C6—C7—H7	120.1	O5—C18—C19	122.41 (13)
C8—C7—C6	119.71 (14)	C17—C18—C19	119.74 (13)
C8—C7—H7	120.1	O6—C19—C18	115.56 (12)
O3—C8—C7	119.61 (13)	O6—C19—C20	124.95 (13)
O3—C8—C9	120.14 (12)	C20—C19—C18	119.49 (13)
C7—C8—C9	120.26 (13)	C15—C20—H20	119.4
O4—C9—C8	114.02 (12)	C19—C20—C15	121.25 (13)
O4—C9—C10	126.09 (13)	C19—C20—H20	119.4
C10—C9—C8	119.89 (13)	O6—C21—H21A	109.5
C5—C10—H10	119.8	O6—C21—H21B	109.5
C9—C10—C5	120.45 (14)	O6—C21—H21C	109.5
C9—C10—H10	119.8	H21A—C21—H21B	109.5
O4—C11—H11A	109.5	H21A—C21—H21C	109.5
O4—C11—H11B	109.5	H21B—C21—H21C	109.5
			
Cl1—C1—C2—O1	−178.22 (12)	C6—C7—C8—C9	0.6 (2)
Cl1—C1—C2—C3	1.4 (2)	C7—C8—C9—O4	179.38 (15)
Cl1—C1—C12—O2	179.55 (11)	C7—C8—C9—C10	−0.5 (2)
Cl1—C1—C12—C13	−0.5 (2)	C8—C9—C10—C5	0.1 (2)
O1—C2—C3—C4	−0.2 (2)	C10—C5—C6—C7	0.0 (2)
O2—C12—C13—C14	2.2 (2)	C11—O4—C9—C8	176.77 (15)
O3—C8—C9—O4	−1.1 (2)	C11—O4—C9—C10	−3.4 (2)
O3—C8—C9—C10	179.02 (15)	C12—C1—C2—O1	1.3 (2)
O4—C9—C10—C5	−179.74 (14)	C12—C1—C2—C3	−179.07 (14)
O5—C18—C19—O6	1.6 (2)	C12—C13—C14—C15	−179.93 (14)
O5—C18—C19—C20	−178.20 (16)	C13—C14—C15—C16	2.7 (3)
O6—C19—C20—C15	179.36 (15)	C13—C14—C15—C20	−177.12 (16)
C1—C2—C3—C4	−179.78 (15)	C14—C15—C16—C17	−178.99 (15)
C1—C12—C13—C14	−177.78 (15)	C14—C15—C20—C19	179.38 (15)
C2—C1—C12—O2	0.0 (2)	C15—C16—C17—C18	0.1 (3)
C2—C1—C12—C13	179.96 (14)	C16—C15—C20—C19	−0.5 (2)
C2—C3—C4—C5	177.85 (15)	C16—C17—C18—O5	178.57 (16)
C3—C4—C5—C6	−2.2 (3)	C16—C17—C18—C19	−1.5 (3)
C3—C4—C5—C10	178.62 (16)	C17—C18—C19—O6	−178.37 (15)
C4—C5—C6—C7	−179.12 (15)	C17—C18—C19—C20	1.8 (2)
C4—C5—C10—C9	179.31 (14)	C18—C19—C20—C15	−0.9 (2)
C5—C6—C7—C8	−0.4 (3)	C20—C15—C16—C17	0.9 (2)
C6—C5—C10—C9	0.1 (2)	C21—O6—C19—C18	−174.18 (16)
C6—C7—C8—O3	−178.88 (15)	C21—O6—C19—C20	5.6 (2)

### 4-Chloro-5-hydroxy-1,7-bis(4-hydroxy-3-methoxyphenyl)hepta-1,4,6-trien-3-one Hydrogen-bond geometry (Å, º)

**Table:**

*D*—H···*A*	*D*—H	H···*A*	*D*···*A*	*D*—H···*A*
O1—H1···O2	0.82	1.70	2.4506 (16)	151
O3—H3···O4	0.82	2.18	2.6395 (15)	115
O5—H5···O6	0.82	2.28	2.7200 (15)	114
O3—H3···O6^i^	0.82	2.20	2.8398 (15)	135
O5—H5···O3^ii^	0.82	2.05	2.8439 (16)	164
C11—H11*B*···O2^iii^	0.96	2.59	3.477 (2)	154
C17—H17···O2^iv^	0.93	2.57	3.3206 (19)	138

Symmetry codes: (i) *x*−1, −*y*+3/2, *z*+1/2; (ii) *x*+1, −*y*+3/2, *z*−1/2; (iii) −*x*+1, *y*−1/2, −*z*+3/2; (iv) *x*, −*y*+3/2, *z*−1/2.
