# 4-Chloro­curcumin. Addendum

**DOI:** 10.1107/S2414314625006352

**Published:** 2025-08-28

**Authors:** Phuong-Truc T. Pham, Mamoun M. Bader

**Affiliations:** a Department of Chemistry Pennsylvania State Scranton Dunmore PA 18512 USA; b Department of Chemistry Alfaisal University,Riyadh 11533 Saudi Arabia

**Keywords:** crystal structure, curcumin derivative, hydrogen bonds, addendum

## Abstract

A reference is added to the article by Pham & Bader [*IUCrData* (2025), **10**, x241243]

## Abstract 2

Addendum to Pham & Bader [*IUCrData* (2025), **10**, x241243].

In the article by Pham & Bader (2025 ▸), a reference to a previous determination of the title compound (Pham & Bader, 2024 ▸) was omitted and is added here.
