# Supplementary material for: 4-Chloro­curcumin
Source: IUCrdata. 2025 Jan 3;10(Pt 1):x241243. doi: 10.1107/S2414314624012434 (PMC11799780; doi:10.1107/S2414314624012434)
Supplement: Supplementary file 3 [file x-10-x241243-sup3.doc]

**Supporting information**

**Pham and Bader IUCr Dec. 10, 2024**

**Table S1.** Summary of main inter and intra molecular interactions chlorocurcumin compared with the most common polymorph of curcumin.

| **Structural Feature** | **Cl-curc** | **Curc**  **Form**  **I** | **Curc**  **Form II** | **Curc**  **Form III** |
| --- | --- | --- | --- | --- |
| Closest O…O *inter*- | 2.840 | 2.666 | 2.625; 2.834 | 2.666  2.975 |
| Closest O…O *intra*- | 2.451 | 2.441 | 2.498  2.527 | 2.490 |
| π-stack distances | 3.362 | NA | 3.306 | 3.817 |
| intra- O-H…O=C | 1.703 | 1.724 | 1.616; 1.676 | 1.724 |
| C-H..π interactions | 2.984 | 2.864 | 3.306; 3.365 | 4.387 |
| Closest Cl…Cl | 5.926 | - | - | - |

**Figure S1.** Structural features used in Table S2.

**Table S2**. Comparison of the main structural features shown in (Figure 4) in title compound compared with three forms of Curcumin

|  | **Chloro-curcu** | **curcufform I**  **P21/n** | **curcu form II**  **Pca21** | **curc**  **form III**  **P b c a** |
| --- | --- | --- | --- | --- |
| O…O *intra* | 2.451 | 2.441 | 2.498 | 2.490 |
| Φ1 | 1.16 | 16.01 | 16.51 | 47.07 |
| Φ2 | 4.14 | 0.85 | 5.07 | 39.87 |
| Φ3 | 4.17 | 12.42 | 8.44 | 0.23 |
| R1 | 1.456 | 1.447 | 1.462 | 1.463 |
| R2 | 1.332 | 1.325 | 1.330 | 1.333 |
| R3 | 1.459 | 1.454 | 1.448 | 1.455 |
| R4 | 1.416 | 1.432 | 1.372 | 1.403 |
| R5 | 1.403 | 1.356 | 1.415 | 1.397 |
| R6 | 1.455 | 1.466 | 1.465 | 1.456 |
| R7 | 1.334 | 1.330 | 1.330 | 1.339 |
| R8 | 1.462 | 1.457 | 1.462 | 1.454 |
| R9 | 1.300 | 1.334 | 1.312 | 1.298 |
| R10 | 1.288 | 1.277 | 1.273 | 1.303 |
| C-Cl | 1.736 | na | na | na |

**Table S3.** DFT calculated properties for chloro curcumin and curcumin

| **Compound** | **E HOMO (eV)** | **E LUMO (eV)** | **Band Gap (eV)** | **Dipole Moment (Debye)** |
| --- | --- | --- | --- | --- |
| Curcumin-H | -5.31 | -1.98 | 3.33 | 4.13 |
| Curcumin- Cl | -5.38 | -2.25 | 3.13 | 1.35 |

**Table S4**. DSC and Spectroscopic Data for Curcumin and α-halo curcumins

| **Compound** | **Melting point (DSC)** | **λ_max_ (CH_2_Cl_2_)** |
| --- | --- | --- |
| curcumin | 184.89 | 401, 421, 442 |
| Curcumin-Cl | 196.71 ^o^C | 427 ,450, 477 |
